# Identification and Characterization of Single-Chain Antibodies that Specifically Bind GI Noroviruses

**DOI:** 10.1371/journal.pone.0170162

**Published:** 2017-01-17

**Authors:** Amy M. Hurwitz, Wanzhi Huang, Baijun Kou, Mary K. Estes, Robert L. Atmar, Timothy Palzkill

**Affiliations:** 1 Interdepartmental Program in Translational Biology & Molecular Medicine, Baylor College of Medicine, Houston, Texas, United States of America; 2 Department of Pharmacology, Baylor College of Medicine, Houston, Texas, United States of America; 3 Department of Molecular Virology & Microbiology, Baylor College of Medicine, Houston, Texas, United States of America; 4 Department of Medicine, Baylor College of Medicine, Houston, Texas, United States of America; US Naval Research Laboratory, UNITED STATES

## Abstract

Norovirus infections commonly lead to outbreaks of acute gastroenteritis and spread quickly, resulting in many health and economic challenges prior to diagnosis. Rapid and reliable diagnostic tests are therefore essential to identify infections and to guide the appropriate clinical responses at the point-of-care. Existing tools, including RT-PCR and enzyme immunoassays, pose several limitations based on the significant time, equipment and expertise required to elicit results. Immunochromatographic assays available for use at the point-of-care have poor sensitivity and specificity, especially for genogroup I noroviruses, thus requiring confirmation of results with more sensitive testing methods. Therefore, there is a clear need for novel reagents to help achieve quick and reliable results. In this study, we have identified two novel single-chain antibodies (scFvs)—named NJT-R3-A2 and NJT-R3-A3—that effectively detect GI.1 and GI.7 virus-like particles (VLPs) through selection of a phage display library against the P-domain of the GI.1 major capsid protein. The limits of detection by each scFv for GI.1 and GI.7 are 0.1 and 0.2 ng, and 6.25 and 25 ng, respectively. They detect VLPs with strong specificity in multiple diagnostic formats, including ELISAs and membrane-based dot blots, and in the context of norovirus-negative stool suspensions. The scFvs also detect native virions effectively in norovirus-positive clinical stool samples. Purified scFvs bind to GI.1 and GI.7 VLPs with equilibrium constant (K_D_) values of 27 nM and 49 nM, respectively. Overall, the phage-based scFv reagents identified and characterized here show utility for detecting GI.1 and GI.7 noroviruses in multiple diagnostic assay formats with strong specificity and sensitivity, indicating promise for integration into existing point-of-care tests to improve future diagnostics.

## Introduction

Norovirus is the leading cause of foodborne illnesses in the United States and is commonly known as the cruise ship or winter bug. Infections spread rapidly and result in roughly 267 million unique cases and 200,000 deaths annually around the globe in addition to significant health and economic repercussions [1–3]. Although evaluation of a candidate vaccine is underway [4,5], there are currently no treatments available for infection beyond rehydration and symptom-directed therapy. Further, the standard diagnostic assays present significant limitations that hinder their ability to identify infection in order to inform effective prevention strategies, especially at the point-of-care. These shortcomings in detection can lead to otherwise preventable outbreaks, while immunocompromised and other especially vulnerable patients who become infected by norovirus can suffer from chronic symptoms and inappropriate treatments [6]. To ameliorate the effects of norovirus in people, developing improved diagnostics can help to prevent outbreaks and inform appropriate clinical responses that will protect sensitive patient populations.

Members of the *Caliciviridae* family of viruses, noroviruses are classified into seven genogroups (GI-GVII) and further divided into genotypes based on the amino acid sequence diversity of the major capsid protein [4,7]. The majority of noroviruses that infect humans appear in genogroups GI and GII, and the first, now prototypical, norovirus identified was Norwalk virus, genotype GI.1 [8]. The norovirus genome is subdivided into three open reading frames—ORF1, ORF2 and ORF3—where ORF2 encodes the major capsid protein, VP1. The surface of the capsid is composed of 180 copies of VP1 arranged into 90 dimers [9]. Each VP1 protein is composed of two major domains—a protruding, P-domain and inner shell, S-domain. Genetic variation and antigenicity across genotypes occurs in the P-domain, which is also the most accessible for binding at the capsid surface [10]. Recombinant virus-like particles (VLPs) purified from a baculovirus expression system demonstrate equivalent antigenic and structural characteristics to native virions and thus are used commonly for laboratory studies [11].

Given the significant limitations in the accessibility, sensitivity and specificity of the diagnostic tools currently in use—including RT-PCR and enzyme immunoassays—there is a clear need for reliable point-of-care tests (POCTs) to rapidly identify and respond to infections by this contagious virus. To date, the only FDA-approved POCT is a lateral-flow immunochromatographic assay by R-Biopharm, called RIDA®Quick. This assay and other POCTs that are available in non-US markets each show fairly high sensitivity for GII noroviruses, particularly GII.4, which makes the tests clinically valuable since GII.4 is currently the most predominant genotype in circulation [12–14]. However, sensitivity for GI noroviruses is very poor overall, with 0% sensitivity for GI.7 in the four commercially available rapid chromatographic POCTs evaluated by Ambert-Balay et al. [15]. Thus, negative results require further confirmation with real-time RT-PCR or sequencing and led the FDA to approve RIDA®Quick only for use during outbreaks and not for individual patient diagnosis [16]. The ability to distinguish between GI and GII infections is also important for epidemiological studies to quantify the incidence and disease burden [17]. Recently, novel detection reagents have been identified by our group, including single-chain antibodies (scFvs), monoclonal antibodies (mAbs), and phage-displayed peptides that can broadly detect GI and GII noroviruses [18–20]. Still, there is a need for reagents that can identify individual genogroups and genotypes specifically and with sufficient sensitivity to contribute to improved POCTs that provide reliable diagnoses and support epidemiological studies.

Given the challenges posed by the current status of norovirus diagnostics, this study aimed to identify novel reagents with specific binding to GI.1 noroviruses with high sensitivity in the context of multiple potential diagnostic formats. Since several existing POCTs detect GII.4 with high sensitivity and specificity [13], we focused on developing reagents that could be included in a cocktail for use ultimately in an assay that detects a broad range of noroviruses with the ability to distinguish between genogroups or even genotypes. Our approach was to use a phage-displayed scFv library to select against the P-domain of GI.1 to increase the likelihood of identifying strong-binding reagents with high sensitivity. Reagents were evaluated using multiple formats including ELISA and solid-phase membrane assays to ensure flexibility in diagnostic utility.

## Materials & Methods

### Modeling the scFv structure

**Fig 1:**
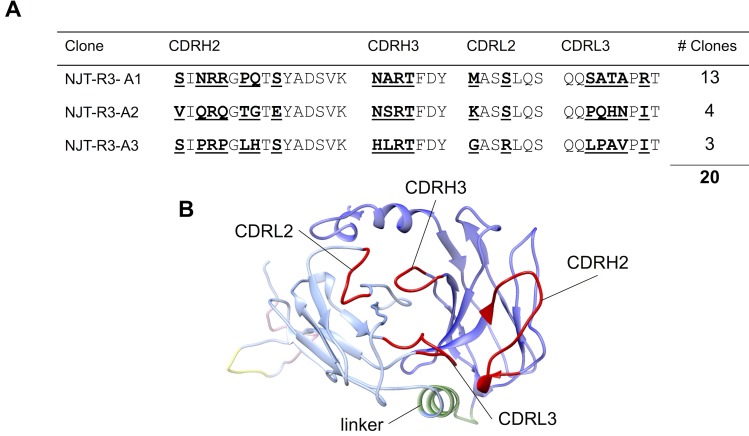
Single-chain antibody sequences and structural model. Three novel phage-displayed single-chain antibodies were identified via biopanning and named NJT-R3-A1, NJT-R3-A2, and NJT-R3-A3 based on their abilities to bind the NV P-domain after three rounds of binding selection **(A)**. The 18 residues randomized in the Tomlinson J library span across four complementarity-determining regions (CDRs) and are indicated in bold and underlined. **(B)** The four CDRs are labeled and highlighted in red on this structural model of the NJT-R3-A3 scFv in which the heavy chain (dark blue) and light chain (light blue) are connected by a linker (green). The scFv protein also contains His6 (yellow) and myc (pink) tags that appear at the back of the model.

The scFv amino acid sequence that is inserted into the pIT2 vector used to produce the Tomlinson J phage library and containing the CDR sequences found in the NJT-R3-A3 clone (Fig 1A) was submitted as a query to the 3D-JIGSAW online server (https://bmm.crick.ac.uk/~3djigsaw/) [21,22]. The server generated a 3D structure of the scFv based on homology modeling.

### Biopanning scFv-displayed peptide libraries

The Tomlinson J phage library (provided by MRC Geneservice) is based on a single human framework for V_Η_ and Vκ containing 18 positions in the CDRH2, CDRH3, CDRL2, and CDRL3 regions randomized with NNK codons to achieve a diversity with ~1.4 × 10^8^ unique clones in a pIT2 vector [21]. For biopanning, purified P-domain from Norwalk GI.1 norovirus was coated directly to immunotubes (Nalgene) in 4 mL of PBS at 5 μg/mL per tube to immobilize overnight at 4°C. Tubes were washed three times with 4 mL of PBS and blocked with 2% nonfat dry milk in PBS (MPBS) for 2 hours while gently rocking at room temperature (RT), followed by three additional washes with PBS. Next, 2.5 × 10^12^ colony-forming units (cfu) of the Tomlinson J library was added to 4 mL of 1% MPBS to incubate in the tubes rotating for 1 hour and standing for 1 hour at RT. After incubation, the immunotubes were washed 10 times with 4 mL of PBS-T (0.1% Tween 20 in PBS). Binding phages were eluted with 0.5 mL of 1 mg/mL trypsin diluted in PBS incubated for 10 minutes rotating, and then transferred to a 1.5 mL microcentrifuge tube. Phages were amplified and titered as described below and used to complete subsequent rounds of biopanning [19]. During rounds two and three of biopanning, 1.5 × 10^12^ cfu of amplified phage from the previous round was used to incubate with P-domain, and 20 washes were performed with PBS-T instead of 10 washes after incubating with phages.

### Phage preparation

#### Phage amplification

*E*. *coli* XL1-blue cells containing a pIT2 vector encoding the desired scFv sequence(s) were grown shaking at 37°C to an optical density of 600 nm (OD_600_) between 0.4 and 0.6. Then, 5 μL of KM13 helper phage was added per 30 mL of culture and incubated for 30 minutes standing still at 37°C. Cultures were then incubated between 20 and 24 hours shaking at 30°C. The next day, cultures were centrifuged for 10 minutes at 5,000 rpm and supernatant was transferred to fresh conical tubes. For every 30 mL of supernatant, 6 mL of PEG6000/2.5 M NaCl were added. The mixture was inverted 100 times and incubated at 4°C overnight. Precipitated phage was centrifuged for 30 minutes at 5,000 rpm and resuspended in PBS.

#### Phage tittering

Purified phages were serially diluted from 10^2^- to 10^8^-fold in *E*. *coli* TG1 cells grown to OD_600_ between 0.4 and 0.6, and incubated on the bench top at RT for 15–30 minutes. Then, 100 μL of each dilution was spread onto LB agar plates containing 100 μg/mL of ampicillin (Amp) and 1% glucose and incubated overnight at 37°C. The number of colonies that grew on each plate was counted to determine the concentration of phages in the prep in terms of cfu/mL. To further evaluate single clones, individual colonies were selected from the titer plates. To determine the sequence of CDR regions of individual scFv clones, cultures were grown from single colonies and DNA was extracted using the Zyppy™ Plasmid Miniprep Kit and sequenced. Cultures were also used to amplify individual phage stocks for further characterization as described below.

### ELISA

Binding signals in all ELISA experiments are reported as the ratio of the average signal produced by a phage-displayed scFv and that of the negative control M13KE phage. Since the average optical density (OD) at 450 nm produced by M13KE phage across all experiments was 0.12 with a standard deviation of 0.05, binding signals that are considered “positive” were required have an OD of at least 0.23, which is the average M13KE signal plus two standard deviations. Positive binding is further defined by a ratio of the signals from scFv and M13KE in matching experimental conditions that is greater than or equal to 2, and where the lower limit of the 95% confidence interval is greater than one.

#### Phage ELISA

Norovirus VLPs were diluted to 5 ug/mL in PBS and coated onto 96-well polystyrene microtiter plates (Immulon HB) at 100 μL/well to incubate overnight at 4°C. All concentrations were tested in duplicate wells. After 3 washes with PBS, wells were blocked with 10% milk in PBS (MPBS) for 2 hours rocking gently at RT. Following 3 washes with PBS, 5 × 10^10^ cfu of phage diluted in 100 μL of 1% MPBS was added to each appropriate well and incubated at RT while gently rocking for 2 hours. Wells were washed 6 times with PBS-T and then incubated with anti-M13 horseradish peroxidase (HRP)-conjugated antibody diluted 1:5,000 in 1% MPBS for 45 minutes shaking at RT. Wells were washed 6 times again with PBS-T, and 100 μL HRP substrate, 3,3’,5,5’-tetramethylbenzidine (TMB) was added to each well to develop for 10 minutes. Finally, 100 μL of stop solution, 1 M H_3_PO_4_ (KPL) was added to each well, and the optical density (OD) was measured at 450 nm on a plate reader (Tecan Infinite M200 Pro).

#### Capture ELISA

96-well polystyrene plates were coated with 100 μL per well of either anti-NV rabbit polyclonal (1:2,500) or monoclonal 3912 (5 μg/mL) antibody [23] in PBS overnight at 4°C. Wells were washed twice with PBS and blocked with 10% MPBS for 2 hours shaking at RT. After another three PBS washes, VLPs were added to wells from 0 to 5 μg/mL in 1% MPBS and distributed into duplicate wells for each condition tested at 100 μL of solution per well. After incubating for 1 hour shaking at RT, wells were washed 3 times with PBS and incubated for 2 hours shaking at RT after addition of 5 × 10^10^ cfu/well of phage. Next, following 6 washes with PBS-T, anti-M13 HRP-conjugated antibody (1:5,000) was added to wells and incubated for 45 minutes shaking at RT. After another 6 washes with PBS-T, wells were developed with 100 μL HRP Substrate for 10 minutes, stopped with 100 μL KPL and measured on the plate reader for OD_450_. As a negative control, 5 μg/mL of bovine serum albumin (BSA) or PBS (indicated by “Ab-only”) was added in place of VLP.

#### Clinical sample ELISA

Stool samples were obtained and evaluated for the presence of norovirus through protocols (H-8390 and H-10690) and consent procedures approved by the Institutional Review Board at Baylor College of Medicine [24,25]. In study H-10690, participants provided written informed consent. In H-8390, informed consent was not obtained and a waiver of consent was approved by the Institutional Review Board; samples were obtained from the clinical laboratory prior to being discarded and were then de-identified. To evaluate the ability of phage-displayed scFvs to detect virus in these samples, microtiter plates were coated in 100 μL of mAb 3912 (5 μg/mL in PBS) overnight, washed three times with PBS, then blocked with 10% MPBS for two hours at room temperature. Stool was dissolved in PBS to produce a 10% stool suspension and serially diluted further down to 0.02%. Stool dilutions were added to microtiter wells with 100 μL per well and incubated for one hour agitating gently at room temperature, followed by 10 washes with PBS-T. Next, 5 × 10^10^ cfu of phage was added to each well and incubated for 2 hours agitating gently at room temperature, followed by another 10 washes with PBS-T. Anti-M13-HRP antibody (1:5,000) was added to wells for 45 minutes, followed by 8 washes with PBS-T. Finally, HRP was developed with TMB reagent for 10 minutes, stopped with KPL, and signals were measured at 450 nm. As a negative control, M13KE phage was used to detect stool at the highest concentration (10% suspension) for each stool sample.

### Dot blot

Either VLPs or clinical stool samples were diluted to the appropriate concentrations and seeded directed onto Whatman nitrocellulose membranes in duplicate 1 μL dots for each dilution. VLPs were diluted in either PBS or 10% negative stool, and positive norovirus stool samples were diluted in PBS. Dots were allowed to dry completely at RT, then blocked with 10% MPBS for 2 hours gently rocking. After three 5-minute washes with PBS-T, phages were added to the membranes at 5 × 10^10^ cfu per mL in 1% MPBS and incubated for 30 minutes on a RT shaker. Membranes were washed again, followed by another 30 minute incubation on the RT shaker with anti-M13 HRP-conjugated antibody (1:5,000) in 1% MPBS. Following three 5-minute washes with PBS-T, TrueBlue™ Peroxidase Substrate (KPL) was added to the membranes for 10 minutes. Membranes were rinsed briefly in distilled water, and imaged with a digital camera. As a positive control, anti-NV polyclonal antibody (1:3,000) was used to detect VLPs in place of phage on a separate blot, and binding was detected with anti-rabbit-HRP conjugated antibody (1:3,000) and developed similarly.

### scFv purification

Phagemid vector, pIT2, containing the desired scFv sequence was transformed into *E*. *coli* RB791 cells and grown overnight at 37°C in 10 mL of 2YT media with 100 μg/mL Amp and 0.1% glucose. One liter of 2YT with 100 μg/mL Amp and 0.1% glucose was then inoculated with 10 mL of the overnight culture and grown to an OD_600_ between 0.8 and 1.0. Next, β-D-1-thiogalactopyranoside was added to a final concentration of 0.4 mM and the culture was incubated for 6 hours at 30°C. Cells were harvested by centrifugation for 15 minutes at 5,000 rpm and cell pellets were re-suspended in 30 mL lysis buffer (50 mM sodium phosphate buffer, pH 8.0, 500 mM NaCl, 10 mM imidazole, 60 μg/mL DNAse, 1 tablet EDTA-free protease inhibitor, 25 mM MgCl_2_). The cell suspension was sonicated in order to obtain whole cell protein lysate, centrifuged for 30 minutes at 10,000 rpm, and the supernatant was filtered through a 0.45 μm Millipore filter. Next, the filtered lysate was loaded onto a 1-mL HisTrap™ FF nickel column (GE Healthcare, 17-5255-01) that was previously equilibrated with wash buffer (50 nM sodium phosphate, 500 mM NaCl, 10 mM imidazole, pH 8.2). The loaded column was then washed with 10 mL wash buffer and another 10 mL with wash buffer containing 20 mM imidazole. Protein was eluted in 1.8 mL fractions with a gradient of imidazole increasing from 0 to 500 mM imidazole on a fast-performance liquid chromatography (FPLC) system. Eluted protein fractions were run on an SDS-PAGE gel to evaluate purity and finally pooled and concentrated.

### Surface plasmon resonance (SPR) analysis of scFv antibody binding affinity

Surface plasmon resonance (SPR) was performed using a Biacore 3000 instrument (GE Healthcare, Sweden). The CM5 sensor chip (GE Healthcare) was used to immobilize mAb 3912 (40 μg/mL) via amine coupling at 25 degrees Celsius with running buffer HBS-P (0.01 M HEPES, 0.15 M NaCl, 0.05% Tween 20, pH 7.4) to a RU level of 10,000. Either GI.1 or GI.7 VLPs were injected (100 μg/mL) at 10 μL/min for 7 minutes and allowed 5 minutes to stabilize. Next, purified NJT-R3-A3 scFvs were injected at various concentrations ranging from 50 to 500 nM at 10 μL/min for 5 minutes followed by 10 minutes to allow for dissociation. Regeneration was achieved between cycles with one injection of 10 mM glycine, pH 2.0 at 20 μL/min for 40 seconds followed by 2 minutes of stabilization. Each cycle began with a fresh injection of VLPs for capture by mAb 3912. As a negative control channel, scFvs were flowed over immobilized mAb 3912 directly in a parallel flow channel without VLPs; the responses from this channel were subtracted from responses observed in the channel with captured VLPs. Kinetic parameters were determined by fitting the resulting curves using the Langmuir model for 1:1 protein binding interactions.

## Results

### Identification of three phage-displayed scFv antibodies that bind GI.1 P-domain

In order to identify antibodies that recognize the P-domain of GI.1 norovirus, the Tomlinson J scFv phage display library was used for biopanning. The Tomlinson J phage library is based on a single human framework for V_Η_ and Vκ containing 18 positions in the CDRH2, CDRH3, CDRL2, and CDRL3 regions randomized with NNK codons to achieve a diversity with ~1.4 × 10^8^ unique clones in a pIT2 vector [21]. Following three progressive rounds of binding enrichment against the P-domain of GI.1, single phage clones were isolated, sequenced, and evaluated for their ability to bind GI.1 VLPs in directly coated and antibody capture ELISAs. Three unique scFvs were identified with the sequences listed in Fig 1A and named NJT-R3-A1, NJT-R3-A2 and NJT-R3-A3. Out of 20 phage clones isolated, thirteen of the clones isolated matched the NJT-R3-A1 sequence, while four matched the NJT-R3-A2 sequence, and three matched NJT-R3-A3. A structural model of an illustrative scFv is shown in Fig 1B, which highlights the locations of the four complementarity determining regions (CDRs) containing 18 total residues that were randomized in the Tomlinson J phage-displayed scFv library. This model was generated by inputting the full-length amino acid sequence of NJT-R3-A3 scFv protein into a protein modeling algorithm through 3D-JIGSAW [22].

**Fig 2:**
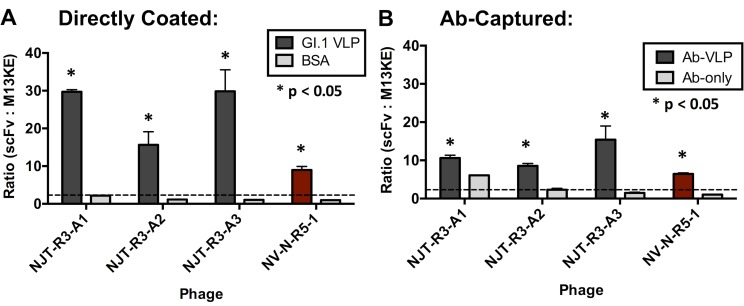
Phage-displayed scFvs detect GI.1 VLPs. Phages displaying the novel single-chain antibodies detect GI.1 VLPs directly coated to the ELISA plate **(A)** and GI.1 VLPs captured by anti-NV polyclonal antibodies **(B)**. As a negative control, phages were also tested for binding against directly coated BSA **(A)** or immobilized antibody with 1% MPBS added for capture without VLPs, labeled “Ab-only” **(B)**. A phage-displayed 12-mer peptide, called NV-N-R5-1 (with sequence LPSWYLAYQKII), known to bind GI.1, is used as a positive control (shown in red) and M13KE phage is used as a negative control for VLP binding. In both ELISAs, 5 × 10^10^ cfu of the appropriate phage was added to detect VLPs in each well. Signals produced by each phage are reported as the ratio with the signals produced by M13KE in the same conditions. Detection of VLP by a given phage is defined as positive when the signal ratio is greater than or equal to two (indicated by the dotted line). Significance is also indicated based on t-tests comparing the signal produced by each phage with VLP and the negative control where p < 0.05. Results in part **(A)** represent the averages and standard deviations from two replicate experiments, and results in part **(B)** are from four replicate experiments.

All three scFv-displaying phages were able to detect GI.1 VLPs effectively in ELISA formats with directly coated VLPs (Fig 2A) and with polyclonal antibody (Ab)-captured VLPs (Fig 2B). Based on the definition of a positive binding signal as described in the Materials & Methods, positive signals were observed for each of the three phage-displayed scFvs detecting VLPs. In addition, all phage samples demonstrated statistically significant binding to VLPs based on Fisher’s t-tests (where p < 0.05), which compare the phage binding to VLPs versus the negative control conditions. None of the three produce significant binding signals in wells containing immobilized BSA, indicating that the positive signals are specific to VLP detection (Fig 2A). Conversely, NJT-R3-A1 showed a positive signal for detecting immobilized antibody without VLPs (‘Ab-only’), which suggests a potential limitation for its utility as a detection reagent due to nonspecific binding and requires additional experiments to further evaluate its specificity for detecting VLPs (Fig 2B). A positive control phage, NV-N-R5-1, first reported by Rogers *et al* [18], shows clear positive signals detecting GI.1 VLPs and no signal against BSA or Ab-only conditions. NV-N-R5-1 is a phage-displayed peptide with sequence LPSWYLAYQKII that was shown to bind specifically to GI.1 VLPs and natural virus. The negative control phage M13KE, which does not display a protein, produced similar OD values in all VLP and negative control conditions, indicating that the three scFvs, and not the phage proteins to which they are attached, are responsible for the positive signals observed.

Although all positive signals produced by the scFvs were strong, on whole, the signals demonstrated when binding to directly coated VLPs were notably higher than those produced when binding to captured VLPs. A possible explanation for this observation could be that the polyclonal capturing antibodies have a limited binding capacity for GI.1 VLPs, resulting in fewer VLPs available for detection by scFvs compared to when VLPs are immobilized directly to the plate. Another explanation could be that the conformation of VLPs differs slightly when they are immobilized versus when they are captured, which results in varied binding affinities. Ultimately, for the purpose of incorporating novel scFvs eventually into clinical diagnostic tools, the experiments moving forward utilized the capture method in order to characterize scFvs in the sandwich ELISA format.

### Specificity of scFv antibody binding

**Fig 3:**
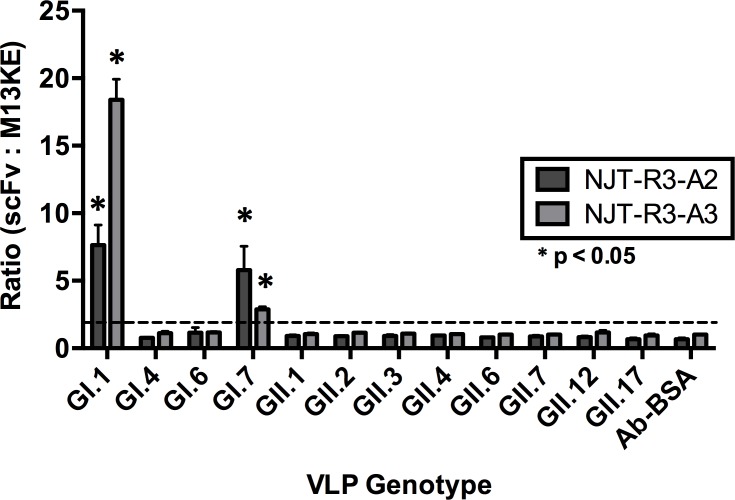
Binding specificity to norovirus genotypes. Binding specificity was evaluated for a panel of norovirus genotypes from genogroups GI and GII. Both NJT-R3-A2 and NJT-R3-A3 phages showed positive binding signals with antibody-captured GI.1 and GI.7, which are defined by signal ratios above 2:1 (labeled with the dotted line). These signals are also statistically significant based on t-tests where p < 0.05. No positive binding signals were observed with any of the GII genotypes tested or the BSA negative control. Each ELISA well received 5 × 10^10^ cfu of the appropriate phage.

After the identification of phage-displayed scFvs and confirmation that they bind GI.1 VLPs, the range of their specificity was determined by evaluating their ability to bind to a panel of antibody-captured VLPs of GI and GII genotypes in order to characterize their potential utility in diagnostic assays. VLPs of each genotype were diluted to 5 μg/mL in 1% MPBS and captured by polyclonal antibodies against NV (GI) or HOV (GII). Both NJT-R3-A2 and NJT-R3-A3 showed positive binding signals for GI.1 and GI.7 VLPs that are also statistically significant based on t-tests comparing the signals for a given genotype against those produced for BSA by the same phage. No significant detection signal was produced against any other VLP genotype tested or for the negative control BSA (Fig 3). Similarly, neither scFv clone was found to detect rotavirus antigen, another virus associated with diarrhea in humans (S1 Fig).

In contrast, NJT-R3-A1 produces lower binding signals overall, with an average optical density of about 0.6, compared to the other two scFvs, and with a ratio greater than 2 with M13KE that is consistent across all genotypes in the panel including the BSA negative control (S2 Fig). This result, combined with those seen in Fig 2B, suggests that NJT-R3-A1 phage does not bind VLPs specifically, but may be additionally binding to the capture antibodies or some other components in the ELISA that results in a moderate level of signal in every condition tested. Thus, NJT-R3-A1 would not be reliable as a detection reagent for identifying virus in stool samples since it would likely produce false-positive results. Moving forward, the functional specificity of NJT-R3-A2 and NJT-R3-A3 were evaluated further to test their abilities to detect norovirus specifically in the context of stool.

**Fig 4:**
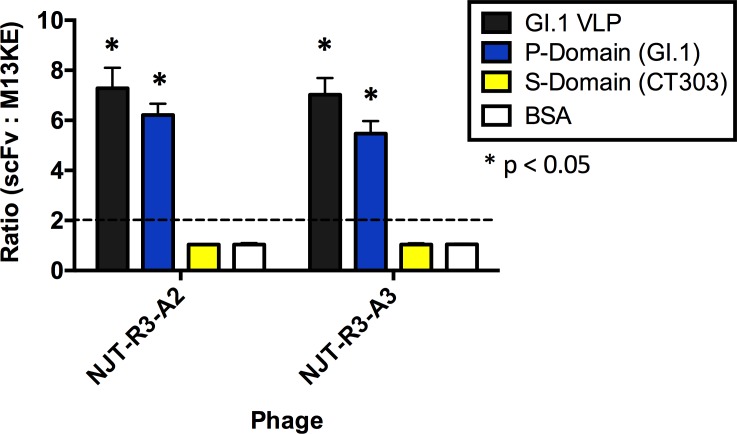
Mapping the binding of phage-displayed scFvs on the major capsid protein. Phage-displayed scFvs bind to the protruding domain (P-domain) of GI.1. Both NJT-R3-A2 and NJT-R3-A3 phages show positive binding signals against the full GI.1 VLP (black) and purified GI.1 P-domain (blue) based on signal ratios above 2 indicated by the dotted line. These signals are also statistically significant based on Fisher’s t-tests when compared to BSA where p < 0.05. Negative signals were seen for the S-domain (CT303) and negative control BSA. Each well contained the same amount of the appropriate phage at 5 × 10^10^ cfu/well.

To understand the interaction between the scFvs and VLPs further, we mapped the binding based on specific domains of the VP1 major capsid protein. Since the scFvs demonstrated strong specificity for GI noroviruses (GI.1 and GI.7, specifically) and had been biopanned originally against the GI.1 P-domain, it was expected that these binding interactions should be occurring largely through the P-domain. To test this hypothesis, an ELISA was performed in which VLPs, P-domain, S-domain (CT303 VLPs that do not express P-domain [26]), and BSA (as a negative control) were each immobilized directly into ELISA wells. The phage-displayed scFvs were used to detect each component. The results indicate that the scFvs interact very strongly and produce positive signals with the GI.1 P-domain in addition to the full GI.1 VLP, while they exhibit no interaction with the S-domain alone (CT303) or BSA (Fig 4).

### Limit of detection for VLPs by phage-displayed scFv antibodies

**Fig 5:**
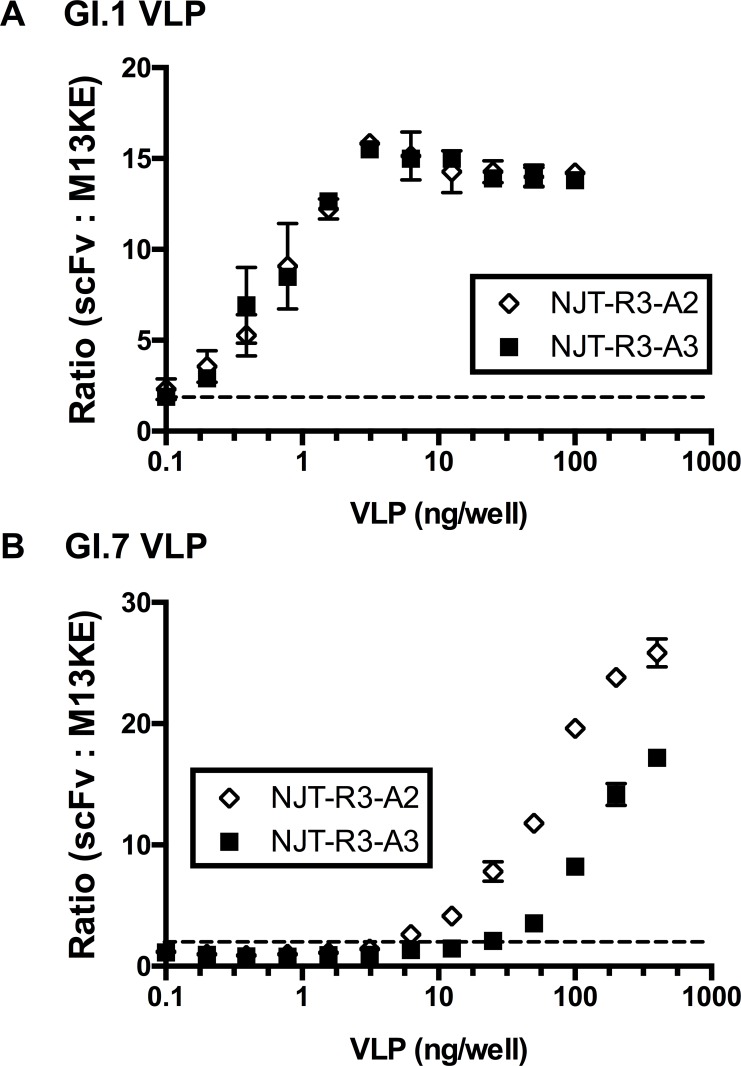
Dose response to GI.1 or GI.7 in 10% negative stool. Phage-displayed scFvs detect GI.1 and GI.7 VLPs specifically in 10% norovirus-negative stool captured by 3912 monoclonal antibodies. VLPs were diluted from a maximum of 400 ng/well to a minimum of 0.10 ng/well. The horizontal dotted line at y = 2 indicates the threshold for positive binding signals based on the definition for positive signals described earlier.

The binding specificity and sensitivity of the scFvs was further investigated by detecting VLPs in the context of stool. In this experiment, GI.1 and GI.7 VLPs were each serially diluted in a 10% norovirus-negative stool suspension from either 400 or 100 ng/well down to 0.10 ng/well and captured by immobilized mAb 3912 for detection by scFvs in an ELISA format. Both phage-displayed scFvs tested—NJT-R3-A2 and NJT-R3-A3—demonstrated the ability to detect GI.1 and GI.7 VLPs with strong specificity, producing positive binding signals in a dose-dependent manner with increasing concentrations of each genotype (Fig 5). No signal was observed when scFvs were added to wells containing stool with no VLPs, indicating that the scFvs do not interact with other components in the stool besides VLPs, which indicates a low likelihood of false-positive results. The negative control phage, M13KE, showed consistently low signals at every VLP concentration, indicating that the scFvs—and none of the other phage capsid proteins—are responsible for binding to VLPs or other artifacts present in the stool.

To evaluate the sensitivity of VLP detection by scFvs, the EC50 and/or limit of detection (LOD) were analyzed for the phage-displayed scFvs. Based on sigmoidal curves fit for the detection of GI.1, the EC50 for NJT-R3-A2 and NJT-R3-A3 are 2.82 ng and 2.10 ng, respectively (Fig 5A). The EC50 for detection of GI.7 could not be determined from the curves since the maximum VLP concentrations tested did not fully saturate the ELISA signal (Fig 5B). Separately, the LOD as defined as the lowest VLP concentration for which the phage produced a significant positive signal, which is a signal greater than or equal to two times the signal produced by M13KE (a threshold indicated by the dotted horizontal lines in Fig 5) where the lower limit of the 95% confidence interval for the ratio of scFv:M13KE signals is greater than 1. The resulting LOD values for detection of GI.1 VLPs diluted in negative stool were 0.1 ng, and 0.2 ng, which converts to 5.9 × 10^6^ and 1.2 × 10^7^ VLP particles, for NJT-R3-A2 and NJT-R3-A3, respectively (Fig 5A). For detecting GI.7 VLPs, the LOD values were 6.25 ng and 25 ng, or 3.7 × 10^8^ and 1.5 × 10^9^ VLP particles, by NJT-R3-A2 and NJT-R3-A3, respectively (Fig 5B). As suggested by the results observed in Fig 3, where signals for GI.1 exceed those elicited for GI.7, these values confirm that the LODs for GI.1 are lower than those for GI.7.

**Fig 6:**
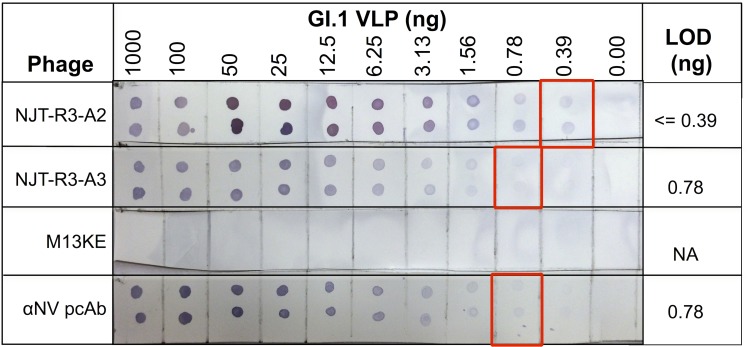
Detection of GI.1 VLPs in dot blot format. Phage-displayed scFvs effectively detect GI.1 VLPs diluted in 10% norovirus-negative stool to amounts smaller than 0.39 ng of VLP per spot (NJT-R3-A2) and 0.78 ng (NJT-R3-A3). As a positive control, anti-NV (GI.1) polyclonal antibody showed dose-dependent positive binding signals to spots with VLPs, while negative control phage M13KE produced no binding signals for any concentration of VLPs or to stool by itself (0 ng VLP).

In addition to the ELISA format, NJT-R3-A2 and NJT-R3-A3 were each evaluated for their ability to detect GI.1 VLPs diluted in stool using dot blots, a second diagnostically relevant format (Fig 6). This provides a proof-of-concept for use of phages in membrane-based immunochromatographic assays, which are commonly used for rapid point-of-care diagnostic applications based on the ease and speed at which they can produce results, including in low-resource settings [27]. VLPs were serially diluted from 1000 ng down to 0.39 ng in 10% norovirus-negative stool and seeded onto nitrocellulose membranes in 1 μL duplicates for each concentration and probed with scFvs for detection. Both phages tested showed clear positive signals that faded gradually towards the lowest VLP concentrations, and no signal for 0 ng VLPs (stool-only). The LOD was determined as the lowest amount of VLPs that elicited a visible signal. For NJT-R3-A2 and NJT-R3-A3, the observed LOD values were less than 0.39 ng and approximately 0.78 ng, respectively. These values are comparable with, or even slightly better than the LOD of 0.78 ng demonstrated by the positive control anti-NV polyclonal antibody tested in parallel. Further, they are consistent with the LODs for GI.1 demonstrated by the scFvs in the ELISA format, which are just slightly lower. As expected, the M13KE negative control phage showed no visible signals for any VLP or stool-only spots. Overall, these results provide evidence that the phage-displayed scFvs would be equally effective reagents in detection assays based on either membrane-based or ELISA formats.

### Phage-displayed scFv antibodies detect virus in clinical stool samples

**Fig 7:**
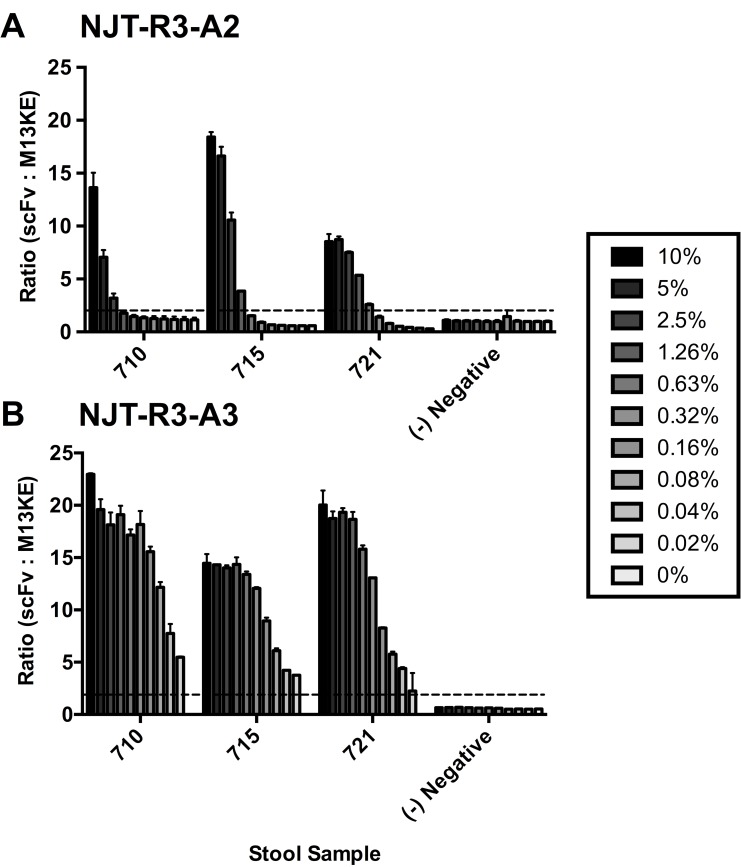
Detection of natural virus in clinical stool samples. Phage-displayed scFvs effectively detect GI.1 virus in clinical stool samples from three patient volunteers, numbered 710, 715 and 721. Stool samples were serially diluted in PBS starting with a 10% stool suspension down to 0.02%. Neither phage-displayed scFv exhibits a positive binding signal with PBS that contains no stool, which is indicated as a 0% stool suspension. Negative control phage M13KE showed little variability in signal, so the signal observed at the highest concentration of each stool sample (10% stool suspensions) was used to determine the signal ratios for every other condition.

In order to determine the utility of these novel phage-displayed scFv antibodies in a clinical diagnostic, their ability to detect virus in clinical stool samples was evaluated. The same ELISA format was used as described previously, in which stool was captured with mAb 3912 and detected with phage-displayed scFvs. Stool from three persons—labeled 710, 715, and 721—collected from a previous challenge study and confirmed positive for norovirus [24] were serially diluted in PBS from 10% to 0.02%, where 0% is PBS-only, and detected with scFvs (Fig 7). Both NJT-R3-A2 and NJT-R3-A3 showed clear, dose-dependent positive signals detecting virus in all three volunteer stool samples, and negative signals for 0% stool (PBS-only) and the norovirus-negative control stool sample. Since the M13KE control phage showed consistently low signals for all stool concentrations, the signal observed for the highest concentration of stool tested (10%) was used to calculate the scFv signal ratios. Altogether, these observations indicate that the two scFvs tested can bind to GI.1 virus specifically in the context of stool samples, indicating great promise for utility in clinical diagnostic applications.

### Purified NJT-R3-A3 scFv binds GI.1 and GI.7 VLPs with high affinity

**Fig 8:**
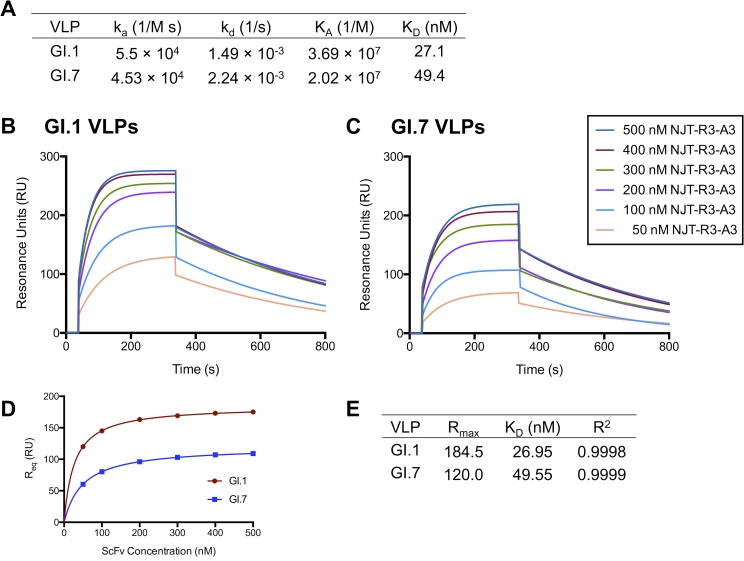
Binding affinity of purified scFv with GI.1 and GI.7 VLPs. Purified NJT-R3-A3 scFvs demonstrate strong binding affinity to GI.1 and GI.7 VLPs captured by mAb 3912. Kinetic parameters **(A)** were determined through surface plasmon resonance studies where 50–500 nM of scFvs were injected over GI.1 **(B)** or GI.7 **(C)** VLPs captured by immobilized mAb 3912. **(D)** To confirm the accuracy of the kinetic parameters, the RU values at equilibrium (R_eq_) in each curve were graphed against their associated scFv A3 concentrations and fit to a hyperbola **(E)**.

Finally, having shown the utility of the phage-displayed scFvs for detecting VLPs and virus in the context of both ELISA and dot blot assay formats, the binding affinity of the NJT-R3-A3 scFv was evaluated. NJT-R3-A3 scFv was chosen based on the strong ELISA signals in the clinical sample detection experiment in Fig 7. NJT-R3-A3 scFv soluble protein was purified and used to bind VLPs captured by mAb 3912 in surface plasmon resonance (SPR) experiments to replicate the format used for virus detection in the earlier ELISAs. Kinetic parameters for binding were assessed based on injections of NJT-R3-A3 antibody at multiple concentrations ranging from 50–500 nM. The resulting binding parameters were determined based on a global fit of the response curves resulting from all of the concentrations using the Langmuir model for 1:1 protein interactions and are shown in Fig 8A, while the curves for binding GI.1 and GI.7 are shown in Fig 8B and 8C [28]. The equilibrium constants (K_D_) were 27.1 nM and 49.4 nM for NJT-R3-A3 scFv binding to GI.1 and GI.7 VLPs, respectively. To confirm these values, the response unit (RU) value at equilibrium (R_eq_) for each curve was graphed against the scFv concentration (Fig 8D) and fit to a hyperbola. The resulting K_D_ values from the curves for GI.1 and GI.7 were 27.0 and 49.6, which match the values from the Langmuir fit very closely (Fig 8E). Thus, the NJT-R3-A3 scFv demonstrates strong binding affinity and specificity to both GI.1 and GI.7 noroviruses.

## Discussion

Improved point-of-care tests (POCT) for detecting norovirus are needed to alleviate the health and economic burdens of infections. While many tests can specifically identify GII.4 with strong sensitivity, the primary limitation with existing diagnostics revolves around the lack of ability to reliably detect and distinguish among the full range of human-infecting strains, including those classified amongst the genotypes appearing in genogroups GI or GII for epidemiological studies [15]. Although the updated RIDA®QUICK immunochromatographic assay (Version N1402) has expanded its specificity to detect some GI genotypes, there is still no evidence that it can detect GI.7 or GI.8 to follow-up on results from the previous version that indicated it could not detect either genotype [15,29]. In addition to having sufficient genotype specificity and sensitivity, the ideal POCT should be easy and rapid to perform and cheap to produce. These qualities would enable individual diagnosis that would inform appropriate treatment of gastrointestinal symptoms, prevention of the rapid spread of outbreaks, and more accurate recording of infections for epidemiological studies. Other diagnostic methods, such as electron microscopy, RT-PCR, and ELISAs are limited by the equipment, expertise and time they require, and thus do not facilitate POC detection. Therefore, improved diagnostic tools are still needed to fulfill the call for an accurate POCT.

In this study, we identified and characterized novel phage-displayed single-chain antibodies (scFvs) that detect GI noroviruses specifically through binding to the P-domain. The identification strategy involved serial biopanning of the Tomlinson J phage library against purified Norwalk GI.1 P-domain to select for binders. Individual clones were picked for sequencing, amplified and evaluated for their ability to bind GI.1 VLPs in the ELISA format. The scFv sequences selected matched one of three identities, which were named NJT-R3-A1, NJT-R3-A2, and NJT-R3-A3. Since NJT-R3-A1 demonstrated non-specific binding, it was not evaluated further for its diagnostic potential for detecting norovirus in stool samples. Conversely, the latter two clones demonstrated highly efficient binding of GI.1 VLPs in both ELISA and dot blot assays. Mapping of the binding interactions with the VP1 norovirus capsid protein indicated that this binding occurred via the GI.1 P-domain. Detection of VLPs was also specific in the context of norovirus-negative stool, resulting in zero false-positive results in stool without VLPs added. Interestingly, in evaluating genotype specificity, both NJT-R3-A2 and NJT-R3-A3 also detected GI.7 VLPs with strong specificity.

Previously, our group has identified other scFvs through biopanning against GII.4 VLPs using the same phage library [19]. Specifically, HJT-R3-A9 and HJT-R3-F7 were found to bind both GI and GII VLPs through interactions with the S-domain and P-domain, respectively. Eluting phages with carbohydrate instead of trypsin also identified additional clones that bind both GI and GII VLPs in ELISAs. Since these biopanning experiments selected for binding with the GII.4 Houston virus (HOV), however, the binding interactions with GII are more sensitive and better characterized than for GI genotypes. Amongst the clones identified, the one that binds GI VLPs best—HJT-R3-A9—had very low signal for GI.7 and did not produce a binding signal with GI.1 VLPs in SPR analyses, though it worked in a sandwich ELISA format [30]. Alternately, HJT-R3-F7, the scFv with the strongest GI.1 binding affinity based on SPR from that study, did not produce significant signals to any of the GI VLPs in the ELISA format, and its clinical utility for binding GI.1 virus in stool was not evaluated.

The work presented in this new study expands our repertoire of scFvs with the addition of strong binders, NJT-R3-A2 and NJT-R3-A3, that each detects GI.1 and GI.7. In contrast to the previous scFvs identified, these exhibit binding specificity for GI only and not GII genotypes, which provides an additional benefit for epidemiological studies that require the ability to distinguish between outbreaks caused by GI and GII strains. Here, we more deeply characterize binding interactions with multiple GI VLPs and virus, and demonstrate diagnostic utility in both ELISA and membrane-based formats. These are also the first scFvs that have strong specificity for GI.7. When combined with other reagents with specificities that cover the other circulating noroviruses and with sufficient sensitivity, these reagents may help to close the gap in the availability of diagnostic tools useful for sporadic cases of gastroenteritis and POCT.

Many monoclonal antibodies have been identified previously that bind to norovirus with a variety of genogroup and genotype specificities. For example, the mAb 3912 used for capturing VLPs and virus in this study was first produced and characterized along with nine additional mAbs against GI.1 VLPs in 1996 [23]. This mAb shares a common, cross-reactive GI binding epitope with mAbs 3901 and 2461 in the P1 subdomain of VP1 [31,32]. Similarly, mAb NS14 exhibits strong genogroup specificity to GII noroviruses [33], and is used in combination with mAb 3912 in commercially available diagnostic assays [34]. Additional studies have identified mAbs with broad specificity for both GI and GII noroviruses, including NV23 [33], 1B4 [35], MAb14-1 [36], and TV20 [37]. Further characterization of several cross-reactive mAbs demonstrated that NV23 has the broadest reactivity and can be used to capture 25 different genotypes from all human-infecting genogroups for detection by HJT-R3-A9 protein in the ELISA format [20]. In the current study, we have reported two scFvs that can provide binding exclusively to GI.1 and GI.7. Therefore, these scFvs would be useful to incorporate into diagnostic assays with existing mAbs to broaden the specificity of existing POCTs, particularly those that aim to distinguish between GI and GII infections.

Ultimately, the development of improved POCTs can ameliorate the significant health and economic burdens caused by norovirus in many ways. Due to its low infectious dose, norovirus infection spreads rapidly across closed populations such as long-term care facilities, schools, and cruise ships [3,38,39]. In such dense communities, rapid identification of the cause of infection would enable appropriate responses such as isolation of infected individuals and decontamination to prevent further spread [13]. In the case of exceptionally crowded living environments, such as the Reliant Park Complex housing >27,000 individuals during Hurricane Katrina in 2005, the availability of rapid diagnostics becomes increasingly essential for infection control [40]. At the individual level, rapid norovirus detection with an approved POCT would enable appropriate care of patients with increased vulnerability, such as transplant recipients, and prevent the administration of inappropriate treatments such as antibiotics that can further complicate symptoms [6,41]. Economically, norovirus costs society approximately $60.3 billion globally every year, including direct healthcare costs and lost productivity [42]. The introduction of effective POCTs would help significantly to reduce this burden by decreasing the total number infections and the cost of care per infection that often add up due to inappropriate clinical responses in the absence of a proper diagnosis [43].

In summary, this study has identified two novel scFvs that effectively bind GI.1 and GI.7 noroviruses with characteristics that make them clinically useful. Future development of an improved POCT for norovirus could incorporate either of these reagents into a cocktail with others that detect separate genotypes and effectively distinguish between GI and GII noroviruses. In addition to immediate diagnostic applications, future structural studies that investigate norovirus binding epitopes can compare these new scFvs to others that have varying binding characteristics in order to identify structural properties that are associated with binding certain genogroups over others. Information from such studies could be used to engineer or evolve new detection reagents with improved sensitivities or alternate specificities for existing or newly emerging norovirus strains. Further work is needed to define the ideal combination of detection reagents to produce the optimal diagnostic POCT followed by clinical evaluation in the field to help prevent and control outbreaks.

## Supporting Information

S1 Fig NJT-R3-A2 and NJT-R3-A3 do not bind to rotavirus. To evaluate the binding potential of the selected scFvs to another viral pathogen associated with diarrhea in humans, an ELISA was performed to detect rotavirus. Wells directly coated with GI.1 VLPs are shown on the left side of the graph (0.625 μg/mL for detection by phage-displayed scFvs or 1.0 μg/mL for detection by M13KE phage in 100 μL PBS), and wells directly coated with rotavirus antigen are shown on the right side of the graph (Crawford SE, et al. *J*. *Virol*. 2006;80:4820–4832). For antigen detection, 2.1 × 10^11^ pfu of the appropriate phage or, as a positive control for rotavirus detection, anti-rotavirus GP511 antibody (1:2000 dilution) was added to each well. Detection of the anti-rotavirus antibody was done with goat a-gp IgG-HRP (1:4000 dilution) in place of anti-M13-HRP antibody used to detect phage. T-test analyses were performed comparing the optical density signals at each condition (performed in duplicates) to those from blank wells, such that p-values below 0.01 indicate significant binding.
(TIFF)

S2 Fig NJT-R3-A1 binds nonspecifically to norovirus VLPs. In an ELISA format where GI and GII VLPs are captured by anti-NV (GI) and anti-HOV (GII) polyclonal antibodies, NJT-R3-A1 shows similar signals for all VLPs tested and the negative control BSA protein. The dotted line at the value of 2 on the y-axis indicates the ratio of signals produced by scFv:M13KE phages above which is considered to be a positive signal.
(TIFF)
