# Supplementary figures and images for: Identification and Characterization of Single-Chain Antibodies that Specifically Bind GI Noroviruses
Source: PLoS One. 2017 Jan 17;12(1):e0170162. doi: 10.1371/journal.pone.0170162 (PMC5240998; doi:10.1371/journal.pone.0170162)

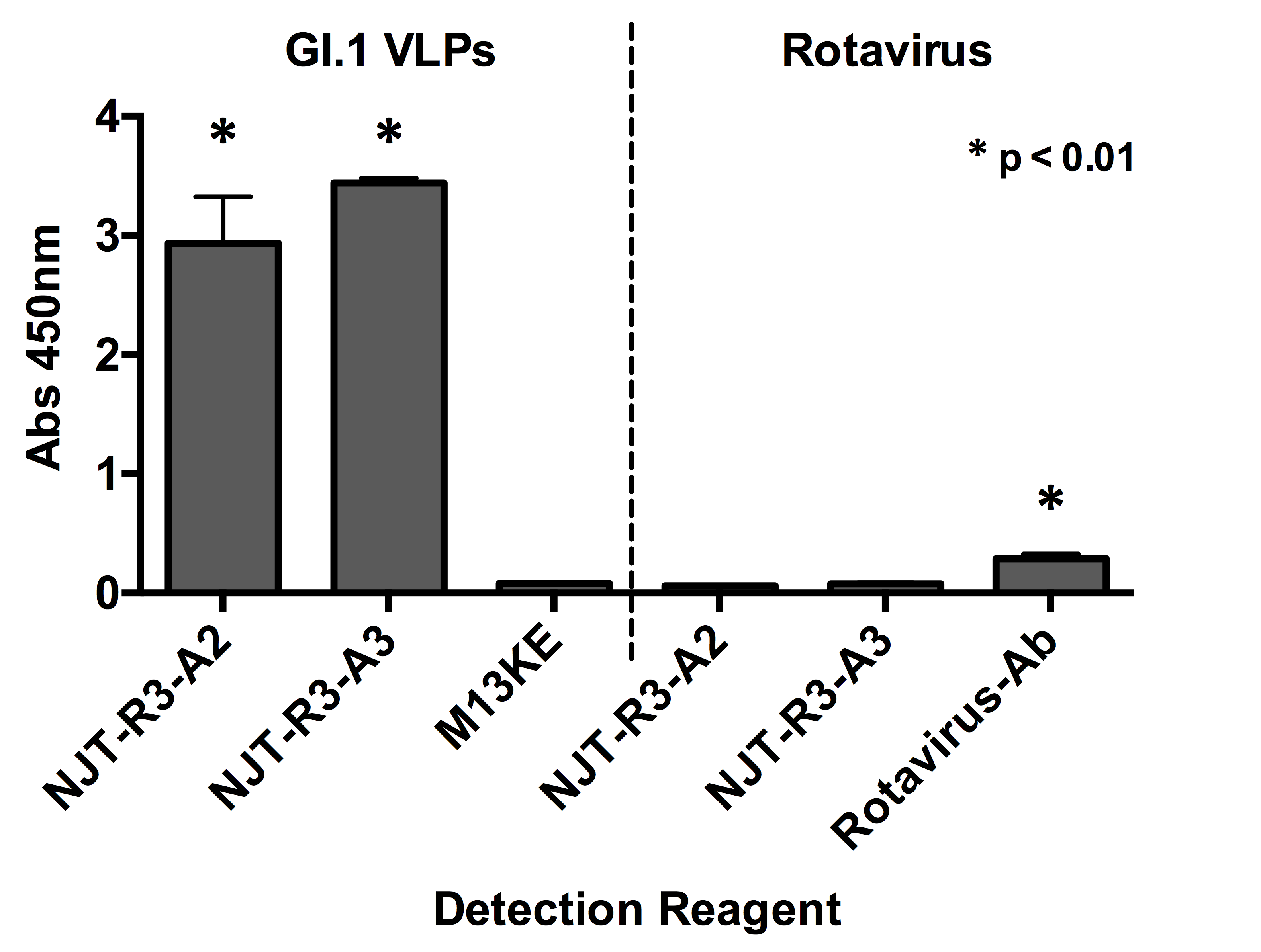

Supplement: S1 Fig — To evaluate the binding potential of the selected scFvs to another viral pathogen associated with diarrhea in humans, an ELISA was performed to detect rotavirus. Wells directly coated with GI.1 VLPs are shown on the left side of the graph (0.625 μg/mL for detection by phage-displayed scFvs or 1.0 μg/mL for detection by M13KE phage in 100 μL PBS), and wells directly coated with rotavirus antigen are shown on the right side of the graph (Crawford SE, et al. J. Virol. 2006;80:4820–4832). For antigen detection, 2.1 × 10^11^ pfu of the appropriate phage or, as a positive control for rotavirus detection, anti-rotavirus GP511 antibody (1:2000 dilution) was added to each well. Detection of the anti-rotavirus antibody was done with goat a-gp IgG-HRP (1:4000 dilution) in place of anti-M13-HRP antibody used to detect phage. T-test analyses were performed comparing the optical density signals at each condition (performed in duplicates) to those from blank wells, such that p-values below 0.01 indicate significant binding. (TIFF) [file pone.0170162.s001.tiff]

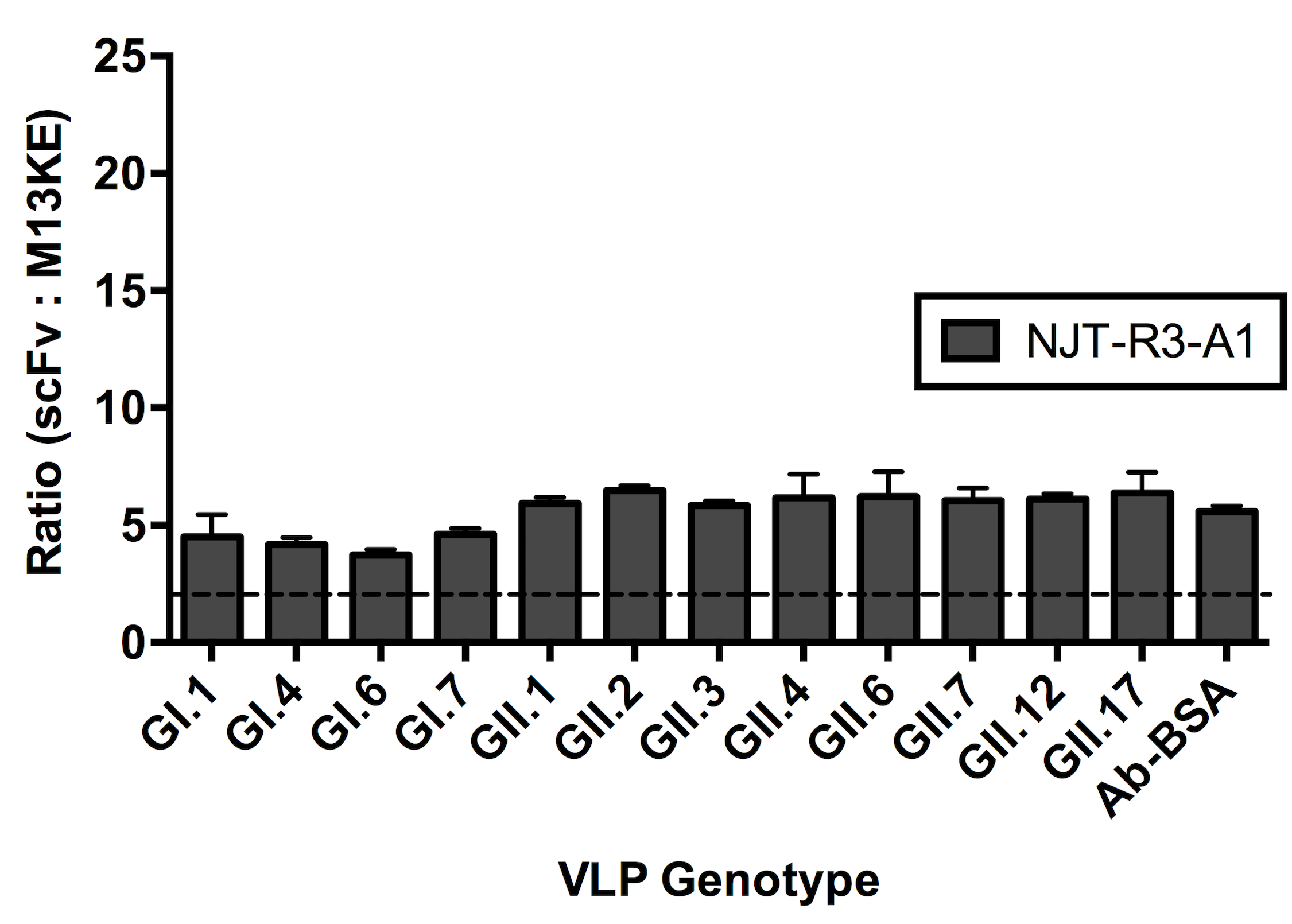

Supplement: S2 Fig — In an ELISA format where GI and GII VLPs are captured by anti-NV (GI) and anti-HOV (GII) polyclonal antibodies, NJT-R3-A1 shows similar signals for all VLPs tested and the negative control BSA protein. The dotted line at the value of 2 on the y-axis indicates the ratio of signals produced by scFv:M13KE phages above which is considered to be a positive signal. (TIFF) [file pone.0170162.s002.tiff]
